# Comparison of different gene-therapy methods to treat Leber hereditary optic neuropathy in a mouse model

**DOI:** 10.3389/fnins.2023.1119724

**Published:** 2023-03-27

**Authors:** Sindhu Velmurugan, Tsung-Han Chou, Jeremy D. Eastwood, Vittorio Porciatti, Yuan Liu, William W. Hauswirth, John Guy, Hong Yu

**Affiliations:** ^1^Bascom Palmer Eye Institute, University of Miami Miller School of Medicine, Miami, FL, United States; ^2^Department of Ophthalmology, College of Medicine, University of Florida, Gainesville, FL, United States

**Keywords:** gene therapy, mitochondrial-targeted, allotopic expression, Leber hereditary optic neuropathy (LHON), mitochondrial-targeted therapy

## Abstract

**Introduction:**

Therapies for Leber hereditary optic neuropathy (LHON), in common with all disorders caused by mutated mitochondrial DNA, are inadequate. We have developed two gene therapy strategies for the disease: mitochondrial-targeted and allotopic expressed and compared them in a mouse model of LHON.

**Methods:**

A LHON mouse model was generated by intravitreal injection of a mitochondrialtargeted Adeno-associated virus (AAV) carrying mutant human NADH dehydrogenase 4 gene (*hND4/m.11778G>A*) to induce retinal ganglion cell (RGC) degeneration and axon loss, the hallmark of the human disease. We then attempted to rescue those mice using a second intravitreal injection of either mitochondrial-targeted or allotopic expressed wildtype human *ND4*. The rescue of RGCs and their axons were assessed using serial pattern electroretinogram (PERG) and transmission electron microscopy.

**Results:**

Compared to non-rescued LHON controls where PERG amplitude was much reduced, both strategies significantly preserved PERG amplitude over 15 months. However, the rescue effect was more marked with mitochondrial-targeted therapy than with allotopic therapy (*p* = 0.0128). Post-mortem analysis showed that mitochondrial-targeted human *ND4* better preserved small axons that are preferentially lost in human LHON.

**Conclusions:**

These results in a pre-clinical mouse model of LHON suggest that mitochondrially-targeted AAV gene therapy, compared to allotopic AAV gene therapy, is more efficient in rescuing the LHON phenotype.

## Introduction

Mitochondrial dysfunction affects almost every tissue in the body, especially those with high energy requirements such as the brain, heart, nervous system, and eye (Desler and Rasmussen, 2012). Leber Hereditary Optic Neuropathy (LHON) is the most common primary genetic mitochondrial disease characterized by losing retinal ganglion cells (RGCs) and optic nerve atrophy (Ellouze et al., 2008). More than 90% of LHON cases are caused by one of the three point mutations in mitochondrial DNA (m. 11778G > A, m.3460G > A, and m.14484 T > C) that encode subunits ND4, ND1, and ND6 of respiration complex I, respectively (Yu-Wai-Man and Chinnery, 1993; Karaarslan, 2019; Zuccarelli et al., 2020).

Pharmacological therapies for LHON, as in most mitochondrial diseases, are inadequate. Gene therapy approaches for LHON have been proposed (Yu et al., 2012; Bacman et al., 2013; Chadderton et al., 2013); However allotopic expression is the only approach that has reached human testing for m. 11778G > A mutation in China (NCT01267422) (Liu et al., 2020), in France (NCT02064569) (Yu-Wai-Man et al., 2020), and in the USA (NCT02161380) (Feuer et al., 2016; Guy et al., 2017; Lam et al., 2022). The French clinical trial is currently in phase 3 trials in the USA and Europe.

The allotopic expression approach involves expressing a nuclear version of a mitochondrial gene in the nucleus and importing the expressed protein back to the mitochondria with the help of the N-terminal fused mitochondrial targeting sequence (MTS) (Koilkonda R. D. et al., 2014; Artika, 2020). Guy et al. (2002) studied the allotopic method extensively in (1) expressing wildtype ND4 in cybrid cells and (2) generating animal models for LHON using mutant human ND4 (R340H) (Qi et al., 2007; Koilkonda R. et al., 2014). Studies proving the use of a second AAV as a vector to carry allotopic wildtype ND4 (Baracca et al., 2005; Qi et al., 2007; Koilkonda R. et al., 2014; Cwerman-Thibault et al., 2015) to rescue RGC degeneration and loss of vision frames the basis for performing gene therapy using allotopically expressed human ND4 in patients carrying m.11778G > A mutation (NCT02161380) (Koilkonda and Guy, 2011; Cwerman-Thibault et al., 2014; Guy et al., 2014, 2017; Koilkonda R. et al., 2014; Feuer et al., 2016; Yu-Wai-Man et al., 2020).

Although visual improvement was observed in patients in those clinical trials, most participants still have low vision, and are still classified as being legally blind, especially for those with the visual loss for more than 1 year (Guy et al., 2014, 2017; Yu-Wai-Man et al., 2020; Yuan et al., 2020). Additionally, the hydrophobicity of ND4 protein limits its ability to cross the mitochondrial membrane and maintain stable long-term gene expression (Oca-Cossio et al., 2003; Perales-Clemente et al., 2011). The entire process, from practical protein synthesis to successful integration into mitochondrial respiration complex, is challenging.

Viruses have the ability to traverse the mitochondrial double membrane to access the inner matrix and deliver DNA inside the organelle (Maul et al., 1978; Kaeppel et al., 2013). Yu et al. (2012) have observed that fusing a MTS to the capsid of adeno-associated virus (MTS-AAV) can redirect the virus to mitochondria rather than the nucleus. When the MTS-AAV-carried *ND4* gene was introduced into the LHON cybrids, ATP synthesis was rescued and when injected into LHON rodents, visual loss and optic atrophy improved post gene therapy. It was also proven using next-generation sequencing that the transferred gene remains episomal, making mito-targeted gene transfer a long-term platform for the treatment of LHON and other primary genetic mitochondrial diseases (Ellouze et al., 2008; Yu et al., 2018).

In this study, we used mito-targeted and allotopic expressed wildtype hND4 to treat LHON mice induced by MTSAAV delivered *hND4G11778A* and compare their therapeutic efficacy to protect the RGCs and axons from dysfunction and degeneration in those mice.

## Materials and methods

### Animals

All animal procedures were performed abiding by the National Institutes of Health Guide for Care and Use of Laboratory Animals and the ARVO Statement for the use of Animals in Ophthalmic and Vision Research. Intraocular injections of recombinant AAV were performed on 3-month-old DBA/1J mice post sedation by inhalation of 1.5–2% isoflurane, right after baseline tests. A local anesthetic (proparacaine HCl) was topically applied to the cornea, and then a 32-gauge needle attached to a Hamilton syringe was inserted through the pars plana. 1 μl of AAVs carrying the gene of interest was injected into the eyes at the interval of 2 days, including MTSAAV/mutant ND4 (4.32 E9 vg/eye), MTSAAV/mCherry (4.52 E8 vg/eye), MTSAAV/wild-type ND4 (4.4 E8 vg/eye), AAV/p1ND4 (4.5 E9 vg/eye), and AAV/Cherry (3.72 E9 vg/eye) as showed in Table 1. For the treatment, the mice were first injected with mutant human ND4, and 2 days later were injected with wildtype human ND4 (Rescued) or m/Cherry (un-rescued). The injection controls received two injections of mCherry. Pattern electroretinograms (PERGs) were performed longitudinally at 3-, 6-, 9-, and 12-month after injections for the allotopic group and baseline, 1-, 3-, 6-, and 12-month after injection for the mito-targeted group.

**TABLE 1:** Summary of experiments.

Study groups	Naïve control	Injection control	Unrescued	Rescued
Mito-targeted (1st)	*n* = 5	MTSAAV/mCherry	MTSAAV/mutND4	MTSAAV/mutND4
		MTSAAV/mCherry	MTSAAV/mCherry	MTSAAV/wtND4
		*n* = 10	*n* = 10	*n* = 10
Allotopic (2nd)	*n* = 10	*n* = 0	MTSAAV/mutND4	MTSAAV/mutND4
			AAV/Cherry	AAV/p1ND4
			*n* = 10	*n* = 10

MTS, mitochondrial targeted sequence; mut, mutant; wt, wildtype; mCherry, mcherry in mito-code.

Cherry, mcherry in standard code.

### Plasmids and AAVs

Plasmids including sc-HSP-ND4G11778A, sc-HSP-wtND4, sc-HSP-mCherry, sc-smCBA-P1ND4, and sc-smCBA-Cherry were constructed as previously described (Koilkonda R. et al., 2014; Yu et al., 2018). In brief, for mito-constructs, human *ND4 G11778A* or wildtype *ND4* gene fused in frame with *FLAG* and mitochondrial-encoded *Cherry*(mCherry) were cloned into scAAV backbones under the control of the mitochondrial heavy strand promoter (*HSP*), where *ND4FLAG* is followed by *mCherry* with a stop codon between two genes (sc-HSP-ND4G11778A or sc-HSP-wtND4). *mCherry* cloned into the same scAAV backbone was used as a control (sc-HSP-mCherry). For allotopic constructs, human *ND4* in standard code was fused with the MTS of ATP synthase subunit C (*p1ND4*) and cloned into scAAV backbones under the control of chicken b-actin promoter (sc-*smCBA-P1ND4*). Also, mCherry in standard code was cloned in the same scAAV backbone and used as a control (sc-*smCBA*-*Cherry*). The resultant plasmids were purified using Qiagen endotoxin free megprep. Then, the mito-constructs were packaged by the University of Florida into MTSAAV2 using VP2COX8, VP1, VP3 (Y444, 500, 730F), and helper plasmid PXX6, and the allotopic constructs were packaged into AAV2 using PDG2mut (Y444, 500, 730F) by National Heart, Lung and Blood Institute’s GTRP AAV facility at Children’s Hospital of Philadelphia (CHOP).

### PERG

Pattern electroretinograms (Chou et al., 2014) were obtained from mice at various time points. In brief, mice were weighed and anesthetized intraperitoneally (IP) using a mixture of ketamine (80 mg/kg body weight) and xylazine (10 mg/kg body weight). A feedback-controlled heating pad was used to maintain the body temperature of individual animals at 37.6°C. PERG signals were recorded from a common subcutaneous needle (Grass Technologies, West Warwick, RI, USA) placed in the snout and referenced to a similar electrode placed in the back of the head. A third subcutaneous electrode placed at the root of the tail served as a ground. Pupils were natural without dilation. A small drop of balanced saline solution was applied topically as necessary to prevent corneal dryness. Visual stimuli consisting of horizontal gratings (95% contrast, 0.06 cycles/degree spatial frequency), 700 cd/m^2^ mean luminance were generated on two (15 cm × 15 cm) LED tablet displays (Jorvec Corp, Miami, FL, USA) and presented at each eye separately at a distance of 10 cm. Gratings reverse in contrast at slightly different temporal frequency (OD, 0.984 Hz; OS, 0.992 Hz) to allow deconvolution of the signal and retrieval of PERG from each eye. PERG signals were fed to an Opti-Amp bioamplifier (Intelligent Hearing Systems Inc., Miami, FL, USA) amplified (10,000-fold), filtered (1–300 Hz, 6 dB/oct), and averaged (OD, 372 epochs of 492 ms; OS, 372 epochs of 496 ms) using a Universal Smart Box acquisition system (Intelligent Hearing Systems Inc., Miami, FL, USA).

### Optic nerve diameter and axon count

A total of 15 months post-injection, optic nerves were dissected from 1 mm behind the ocular bulbs. For quantifying the axon counts, transmission electron micrographs were photographed by a masked observer at a magnification of 1500X for each optic nerve specimen (*n* = 3 in each group). The diameter and number of axons were manually counted by a masked observer. In the mito-targeted group, a total of 2,226 axons were counted for the un-rescued group, 3,049 axons for the rescued group, and 3,320 axons for mcherry double injected control group. In the allotopic group, a total of 4,695 axons were counted for the un-rescued group, 6,445 axons for the rescued group, and 5,229 axons for the I group.

### Statistical analysis

GraphPad Prism software was used to perform univariate statistical analysis. To compare PERG amplitude changes over time between controls and treated mice, the method of Generalized Estimating Equations (GEE) was used (IBM SPSS statistics Ver. 26). GEE is an unbiased non-parametric method to analyze longitudinal correlated data, accounting for the inclusion of both eyes in the design. In the analysis, PERG amplitude was the dependent variable, and age at testing period and treatment group (Controls, Treated) were predictor variables. Main effects (Age, Group) and interaction between age and treatment group were computed, as well as pairwise comparisons between age and treatment group. PERG latency tended to increase with age in all groups. As the differences between groups were relatively small, latency changes were not specifically analyzed but were included as covariates. *P*-values of < 0.05 were considered statistically significant. Values were expressed as means ± standard deviation (SD).

## Results

### Mito targeted wildtype ND4 mediates a rapid and more efficient vision rescue than allotopic expressed ND4

To compare the therapeutic efficacy of the mito-targeted- and allotopic- gene therapy, we performed two experiments in which a LHON mouse model was first generated by intravitreal injection of MTSAAV carrying mutant human *ND4G11778A*(*mutND4*) and then rescued using a second injection of either mito-targeted or allotopic wildtype human *ND4* (wtND4) (Table 1). The mito-targeted *wtND4* was in mitochondrial genetic code, while, allotopic expressed *wtND4* was recoded in nuclear (standard) code and fused in-frame with MTS of the ATP synthase subunit C (p1ND4) (Guy et al., 2002). Unrescued mice received a second injection of *mCHERRY* encoded in either mitochondrial (mito-targeted) or standard (allotopic) genetic code. Both experiments included a group of non-injected mice as naive controls. The rescue efficacy of the two strategies was assessed using longitudinal PERGs, a sensitive electrophysiological measure for RGC function *in vivo*.

**FIGURE 1:**
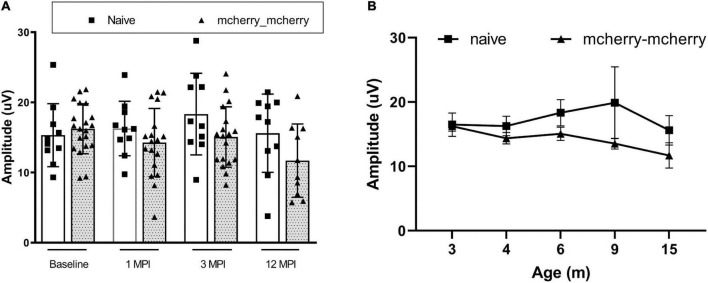
Double injection does not impair the RGC function. **(A)** Scatterplot of PERG amplitudes (average of both eyes) of all mice tested in each group shows no significant difference between naïve and mcherry-double injected mice from baseline, 1-, 3-, and 12-month post-injection. **(B)** A line graph shows the mean PERG amplitude of the mice in both groups did not decrease significantly with age from 3 to 15 months after birth (from baseline to 12 months after injection).

To detect if double injections *per se* impaired the RGC function, we compared the PERG amplitude between naïve and mcherry_mcherry double injected mice from baseline to 1-, 3-, and 12-month after injection (Figure 1A). No significant decrease was found in PERG amplitude with age for both naïve and mcherry double- injected mice (*P* = 0.096). We did not find a statistically significant difference in PERG amplitude between the two groups (*P* = 0.082) or any interaction between age and group (*P* = 0.322) (Figure 1B).

**FIGURE 2:**
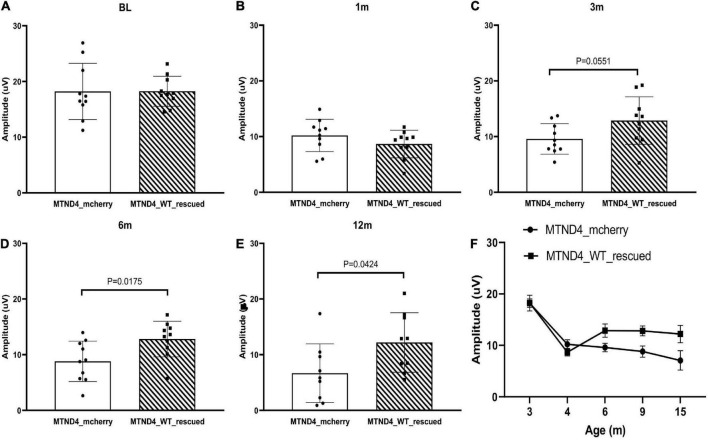
Mito-targeted *wtND4* rescue mutND4-induced RGC dysfunction. **(A)** Scatterplot of PERG amplitudes (average of both eyes) of all mice tested in each group showed no significant difference at the baseline (BL) between un-rescued (MTND4_mcherry) and rescued mice (MTND4_WT_rescued). **(B)** Mutant ND4 injection induced a significant decrease in PERG amplitude at 1 month after injection. Wildtype ND4 reverse the reduction PERG from 3 months **(C)**, 6 months **(D)**, to 12 months **(E)** after injection. **(F)** A line graph shows the mean PERG amplitude for un-rescued mice significantly decreased with age relative to rescued mice from 6 to 15 months after birth.

Next, we wanted to detect whether mito-targeted *wtND4* rescued the RGC dysfunction induced by *mutND4* in the injected mice. Compared to age matched un-rescued control mice (MTND4_mcherry), mice in both groups had a similar baseline PERG amplitude before injection (Figure 2A); however, a significant decrease in PERG amplitude was found at 1 month after injection in both groups (Figure 2B). The rescued mice (MTND4_WT_rescued) regained the PERG amplitude at 3 months (*p* = 0.055, Figure 2C), and the difference between the rescued and unrescued mice became statistically significant at 6 (*p* = 0.018, Figure 2D) and 12 months (*p* = 0.042, Figure 2E) after injection. GEE analysis showed a significant change starting at the age of 6 months (3 months after injection) and persisted to 9 (6 months after injection) and 15 months after birth (12 months after injection). The mean PERG amplitude significantly decreased with age (<0.001); specifically, the effect between the two groups was statistically significant (*p* = 0.039), suggesting mito-targeted wildtype ND4 efficiently reverts RGC loss in LHON mice (Figure 2F).

**FIGURE 3:**
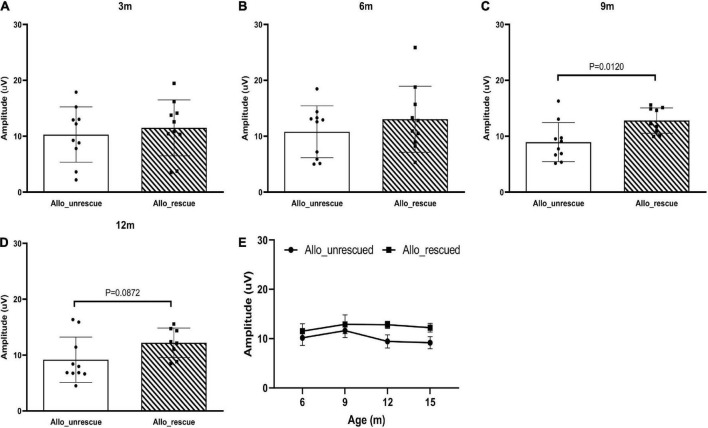
Allotopic expressed wtND4 rescue mutND4-induced RGC dysfunction. Scatterplots of PERG amplitudes (average of both eyes) of all mice tested in each group showed PERG amplitude of unrescued (Allo–unrescue) and rescued (Allo_rescue) mice at 3 months **(A)**, 6 months **(B)**, 9 months **(C)**, and 12 months **(D)** post-injection. **(E)** A line graph shows the mean PERG amplitude for un-rescued mice significantly decreased with age relative to rescued mice from 9 to 15 months after birth.

Then, we wanted to see if the allotopically expressed wtND4 rescued RGC dysfunction in the injected mice. For this experiment, data were available for the post-injection period (3, 6, 9,12 months of age, 6, 9, 12, 15 months after injection) as a confirmation of our previous studies using allotopic vector in the same mouse strain of the same age range (Koilkonda R. et al., 2014; Koilkonda R. D. et al., 2014). Compared to unrescued mice (Allo-unrescued), the rescued mice (Allo-rescued) showed an overall increase in PERG amplitude of about 15% (*P* = 0.017) that was more marked in 9–12 months after injection (Figures 3A–E), suggesting that allotopic expressed wtND4 mediates a moderate and delayed rescue in LHON mice.

**FIGURE 4:**
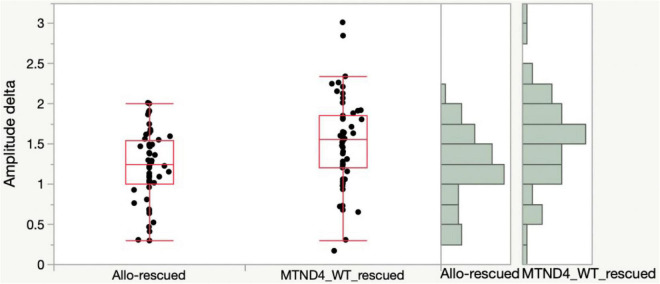
Mito-targeted approach mediates a stronger rescue than allotopic strategy. PERG amplitude differences between rescued and unrescued mice are different from zero for both allotopic and mito-targeted approaches (both are effective in rescue) but the rescue effect is stronger with the mito-targeted approach (the distributions are significantly different for the two approaches, *p* = 0.0128).

Lastly, to determine if any difference exists in rescue effects of RGC dysfunction in LHON mice between mito-targeted and allotopic gene therapy, data was analyzed as mean PERG amplitude change between the rescue group and its corresponding non-rescued control (MTND4_WT_rescued and MTND4_mcherry vs. Allo-rescued and Allo-unrescued). As no baseline PERG data were collected for the allotopic group, rescue-induced PERG changes could not be compared relative to baseline. However, we were able to analyze rescue effects by comparing post-injection data with corresponding post-injection data of unrescued mice. Namely, we subtracted individual rescued PERG amplitudes from the grand mean of corresponding unrescued data over a comparable age ranges (3, 6, 12 MPI). Kolmolgorov-Smirnoff tests showed that the distributions of both allotopic and mito-targeted approach data points were different from zero, indicating both strategies were effective in rescue; however, the rescue effect was more robust with the mito-targeted approach (*p* = 0.0128) (Figure 4).

### Mito targeted wildtype ND4 mediates a more efficient rescue of axons in the optic nerve compared to allotopic expressed ND4

**FIGURE 5:**
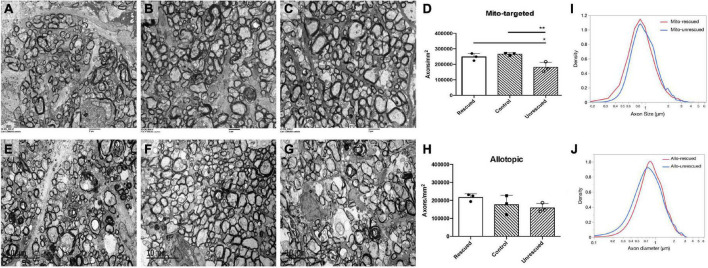
Wild type ND4 protect optic nerve degeneration. Transmission electron micrographs of the retrobulbar optic nerve were performed 15 months post-injection. More cystic spaces are observed in un-rescued mice **(A,E)** when compared to mcherry double-injected control **(B)**, mito-targeted ND4 rescued **(C)**, naïve **(F)**, and allotopic expressed ND4 rescued **(G)** mice. Quantification of axons in the mice of mito-targeted **(D)** and allotopic **(H)** rescue experiments. Only mito-targeted ND4 significantly preserved axons compared to its corresponding non-rescued controls. In contrast, allotopic expressed ND4 did not. Analysis of the distribution of axonal diameters showed that mito-targeted ND4 **(I)** preserved small axons with a shift to larger diameter axons in the corresponding non-rescued compared to the allotopic group **(J)**.

To evaluate the rescue efficacy of optic atrophy induced by mutant ND4 in mice, we performed post-mortem ultrastructural analysis 15 months after intravitreal injections. The optic nerves of the mutant *ND4* injected untreated mice (MTND4_mcherry, Figure 5A) had many cystic spaces and electron-dense debris where axons were degraded. In contrast, age-matched control (mcherry_mcherry, Figure 5B) and mito-targeted *ND4* treated mice (MTND4_WT_rescued, Figure 5C) exhibited numerous axons. Quantitative analysis revealed 33% axons loss in untreated mice compared to controls (186995 ± 33784 vs. 278898 ± 14203, axons/mm^2^, *p* = 0.009), while wildtype *ND4* treatment increased the axons by 37% (256132 ± 22452, axons/mm^2^, *p* = 0.033, Figure 5D). Consistently, the optic nerves of unrescued mice (Allo-unrescue, Figure 5E) in the allotopic group also had more cystic spaces compared to naïve (Figure 5F) and rescued mice (Allo-rescue) (Figure 5G). Axon quantification revealed an increase of 37.5% in Allo-rescue mice (216577 ± 20351 vs. 157768 ± 25500, axons/mm^2^) compared to unrescued mice (Figure 5H). Although the sample size for the axon density measurement was small (*n* = 3 per group), there was a significant difference between mice rescued with the mito-targeted strategy (*p* = 0.0095) compared to mice rescued with the allotopic strategy (*p* = 0.158).

To compare the distribution of axons diameters in unrescued and rescued LHON mice, we manually measured the diameter of every axon that was imaged as described in the methods. The proportion of axons of different diameters was calculated relative to the total existing axons counted for each eye. Compared to age-matched unrescued mice, the axon distribution of MTND4-rescued mice tended to shift toward small axons (Figure 5I), whereas no apparent shift was evident in Allo-rescued mice (Figure 5J). The data suggests that mito -targeted delivery systems for gene therapy may preserve small axons that are preferentially lost in human LHON (Sadun et al., 2000).

## Discussion

Mammalian mtDNA is present as thousands of copies per cell in most cells and mutations can occur in all (homoplasmy) or a fraction of them (heteroplasmy) (Frazier et al., 2019; Naeem and Sondheimer, 2019; Jackson et al., 2020; Filograna et al., 2021). Virtually all pathogenic mutations in mtDNA are functionally recessive (Filograna et al., 2021), including mutant ND4 (m.11778G > A). The impact of pathogenic mutations will not be biochemically and clinically manifest until the ratio of mutated to wildtype mtDNA exceeds a certain upper limit (Shoffner et al., 1990; Rossignol et al., 2003; Falabella et al., 2022). A load of heteroplasmy, defined as the relative amount of mutated to wildtype mtDNA, corresponds to timing and symptom severity (Naeem and Sondheimer, 2019; Jackson et al., 2020). Thus, shifting the heteroplasmic equilibrium without correcting all, or perhaps even most mtDNA mutations, can lead to a shift in disease onset and symptom severity due to complementation of wildtype molecules for the effects of the mutation (DiMauro and Schon, 2003; Desquiret-Dumas et al., 2012; Naeem and Sondheimer, 2019; Jackson et al., 2020). Heteroplasmic shifting can be induced at different levels of mitochondrial gene expression, such as the Mito-targeted strategy induces the shift at DNA level while allotopic strategy induces at protein level.

This study compared rescue effects of two gene-therapy strategies—allotopic and mito-targeted—using a LHON mouse model induced by MTSAAV-delivered *hND4G11778A*. RGC function was investigated in response to coarse patterns of high-contrast (0.05 c/deg = 2.77 logMAR) (Yu et al., 2012; Chadderton et al., 2013; Liu et al., 2020) to detect severe visual deficits that are also characteristic of human LHON and potential reversal of visual loss after gene-therapy. We found that both allotopic and mito-targeted gene therapy strategies were effective in improving visual function compared to age-matched unrescued mice. However, the rescue effect of mito-targeted gene therapy appeared to occur earlier than that of allotopic strategy.

Mitochondria retain only a small number of genes, which encode highly hydrophobic proteins throughout evolutionary history (Johnston and Williams, 2016). One of the most common hypotheses about this phenomenon is the difficulty of importing and sorting those proteins across the mitochondrial membrane if produced remotely (Adams and Palmer, 2003; Allen, 2015; Johnston and Williams, 2016). The allotopic approach delivers the *ND4* gene in standard genetic code into the nucleus and then translocates the expressed ND4 protein from cytosol back to mitochondria. In contrast, mito-targeted strategy delivers *ND4* gene directly inside mitochondria using a mitochondrial targeting AAV (MTSAAV), and the ND4 protein is expressed inside mitochondria, making this strategy more likely to have a higher delivery efficacy and an earlier rescue than the allotopic approach.

The difference in rescue between these two strategies is in agreement with our previous observation. An allotopic test article, produced by the University of Florida, initiated significant rescue 12 months after injection in the same LHON mouse model as in this study (Koilkonda R. et al., 2014). Instead, mito-targeted wildtype ND4 mediated a marked reversal of visual function loss in mutant ND4 transgenic mitomice started from 1 month and sustained up to 12 months after injection (Yu et al., 2018).

Delayed therapeutic effects of the allotopic vector were also evident in the clinical trials for LHON. In the clinical trial we performed, a substantial improvement of > = 15ETDRS (Early Treatment Diabetic Retinopathy Study) letters was noted from 12- to 24-month post-injection (Feuer et al., 2016; Guy et al., 2017; Lam et al., 2022). Similarly, the French Gensight study group reported that a mean (SD) improvement in BCVA of −0.308 (0.068) LogMAR, equivalent to a gain of 15 ETDRS letters for changes from the baseline, was evident at 96 weeks after the treatment with the allotopic expressed ND4 in their phase 3 clinical trial (Yu-Wai-Man et al., 2020; Newman et al., 2021).

Rapid rescue is clinically relevant in treating LHON patients, especially for those who have bilateral simultaneous onset of acute visual loss or unilateral cases with acute visual loss in one eye 6–8 weeks before vision loss in the second eye. Rapid rescue of RGCs using gene therapy might prevent optic nerve degeneration or prevent visual loss in the second eye, even though oxidative injury and apoptosis may already be irreversible at this time. Also, the enhanced survival of RGCs might mediate vision improvement in both eyes as we have observed in the current LHON clinical trials where vision improved bilaterally with unilateral gene therapy injection.

In conclusion, our data shows that the severe visual loss induced by a mitochondrial disease may be reversed for most of the lifespan of laboratory mice using both mito-targeted and allotopic expressed gene therapy. However, mito-targeted therapy likely mediates a quicker and more efficient rescue than the allotopic strategy. This study has certain limitations, such as the experiments were performed in different time periods and under conditions that were similar but not identical, including the test articles that were produced from different facilities. The allotopic vector was made in a GMP lab using two plasmids for the package, while the mito-target vector was produced in a non-GMP lab using five plasmids for the package. This might induce some difference in the titer, purity, and empty/full capsid ratio of the two vectors. Besides, the mouse number, gender, and gene therapy protocols used to rescue the animals were similar within a protocol but not identical among different groups. Further investigations will be needed to confirm the findings. Still, this study provides preliminary experimental evidence supporting mito-targeted gene therapy as a long-term platform for treating human mitochondrial optic neuropathies such as LHON.

## Data availability statement

The original contributions presented in this study are included in the article/supplementary material, further inquiries can be directed to the corresponding authors.

## Ethics statement

The animal study was reviewed and approved by the Institutional Animal Care and Use Committee, University of Miami.

## Author contributions

JG and HY designed the research. SV, T-HC, JE, YL, and HY performed the research. WWH contributed to new reagents/analytic tools. SV, T-HC, VP, and HY analyzed the data. SV, VP, and HY wrote the manuscript. All authors contributed to the article and approved the submitted version.
